# Supplementary material for: From local resynchronization to global pattern recovery in the zebrafish segmentation clock
Source: eLife. 2021 Feb 15;10:e61358. doi: 10.7554/eLife.61358 (PMC7984840; doi:10.7554/eLife.61358)
Supplement: Supplementary file 1. [file elife-61358-supp1.docx]

**Supplementary File 1 Parameter values used in Figs. 2 and 3**

|  | tissue geometry |  |
| --- | --- | --- |
| parameter | **description** | **value** |
| *L_x_* | tip of the tailbud | 385 μm |
| *r* | PSM radius | 25 μm |
| *R* | radius of the half torus for the tailbud | 60 μm |
| *X_c_* | *x* position of the center of the half torus | 300 μm |
| *d_c_* | cell diameter | 11 μm |
| *s_r_* | magnitude of radius change in Eq. (15) | 0 μm min^–1^ |

|  | cell advection Eq. (2) |  |
| --- | --- | --- |
| *v_a_* | advection speed at the anterior end of the PSM | 1.67 μm min^–1^ |
| *v_p_* | strain rate at the posterior domain | 3 μm min^–1^ |
| *x_q_* | *x* position at which the strain rate changes | 0.3 |
| *t_g_* | time at which the advection pattern changes | - |

|  | cell movement and intercellular force Eqs. (3)-(9) |  |
| --- | --- | --- |
| *v_s_* | maximum cell movement speed at the tip of the tailbud | 1.0 μm min^–1^ |
| *X_v_* | normalized length scale of cell mobility gradient | 0.4 |
| *h* | steepness of cell mobility gradient | 3 |
| *D_φ_* | polarity noise intensity | 0.026 min^–1^ |
| *μ* | intercellular force coefficient | 8.71 μm min^–1^ |
| *μ_b_* | boundary force coefficient | 20 μm min^–1^ |
| *r_b_* | length scale of boundary force | 1 μm |

|  | phase equation Eqs. (10)-(12) |  |
| --- | --- | --- |
| ω_0_ | autonomous frequency at the tip of the tailbud | 0.2094 min^–1^ |
| $\sigma$ | difference in the frequency between anterior and posterior ends of the PSM | 0.66 |
| *k* | shape parameter for frequency profile | 3.07 |
| *D_θ_* | phase noise intensity | 0.0013 min^–1^ |
| *κ_s_* | rate of the increase in coupling strength | 0 min^–2^ |
| *κ*_0_ | offset of the coupling strength | 0.07 min^–1^ |
| *T_a_* | period at the anterior end of the PSM *x_a_* | 30 min |

|  | PSM shortening Eqs. (13), (14) |  |
| --- | --- | --- |
| *u_a_* | speed of PSM shortening | 0 μm min^–1^ |
| *S* | segment size | 50 μm |
| *c* | sum of *u_a_* and *v_a_* | 1.67 μm min^–1^ |
| *ρ*_0_ | cell density | 0.0015 μm^–3^ |
| *ζ* | anterior limit of cell addition | 100 μm |
