# Supplementary material for: From local resynchronization to global pattern recovery in the zebrafish segmentation clock
Source: eLife. 2021 Feb 15;10:e61358. doi: 10.7554/eLife.61358 (PMC7984840; doi:10.7554/eLife.61358)
Supplement: Supplementary file 2. [file elife-61358-supp2.docx]

**Supplementary File 2 Parameter values used in Fig. 4 and figure supplements**

| Fig. 4 | (A) All the parameter values are the same as in Sup. File 1.  (B) *L_x_* = 720, *X_c_* = 635, *u_a_* = 0.66, *v_a_* = 1.01, and ω_0_ = 0.2001. Values of all the other parameters are the same as in Sup. File 1.  (C) *t_g_* = 270 min, *v_p_* = 0.16 for *t* < *t_g_* and *v_p_* = 3 for *t* ≥ *t_g_*. Values of all the other parameters are the same as in Sup. File 1.  (E), (F) *L_x_* = 720, *X_c_* = 635. *u_a_* = 0.66, *v_a_* = 1.01 and ω_0_ = 0.2001. *t_g_* = 240 min, *v_p_* = 0.16 for *t* < *t_g_* and *v_p_* = 3 for *t* ≥ *t_g_*. *κ_s_* = 0.06/780, and *κ*_0_ = 0.035. Values of all the other parameters are the same as in Sup. File 1. |
| --- | --- |
| Fig. 3-fig. sup. 4 | (A)-(C) *v_s_* = 0.2-1.6. Values of all the other parameters are the same as in Sup. File 1. |
| Fig. 3-fig. sup. 5 | (A)-(C) *L_x_* = 300, 400, 500, 600. *X_c_* = 215, 315, 415, 515. All the other parameters are the same as in Sup. File 1. |
| Fig. 3-fig. sup. 6 | (B) *v_p_* = 0.16 for the left panel, *v_p_* = 3 for right panel. (C) *v_p_* = 0.16 – 4.42. All the other parameters are the same as in Sup. File 1. |
| Fig. 3-fig. sup. 7 | *r* = 15 - 30. All the other parameters are the same as in Sup. File 1. |
| Fig. 3-fig. sup. 8 | *κ_s_* = 0 and *κ*_0_ = 0.04 - 0.13. All the other parameters are the same as in Sup. File 1. |
| Fig. 3-fig. sup. 9 | (A)-(D) *κ_s_* = *κ*_0_ = 0. *D_θ_* = 0, 1.3×10^–3^, 2.6×10^–3^. All the other parameters are the same as in Sup. File 1. (E) *κ_s_* = 0, *κ*_0_ = 0.07. *D_θ_* = 0, 1.3×10^–3^, 2.6×10^–3^. All the other parameters are the same as in Sup. File 1. |
| Fig. 3-fig. sup. 10 | *k* = 0.5, 3.0, 5.5, 8.0, 10.5. All the other parameters are the same as in Sup. File 1. |
| Fig. 3-fig. sup. 11 | *R* = 30 - 80. All the other parameters are the same as in Sup. File 1. |
| Fig. 4-fig. sup. 1 | *L_x_* = 720, *X_c_* = 635. For *u_a_* = 0.66, *v_a_* = 1.01 and ω_0_ = 0.2001 in (A), (B), (C), and (F). For *u_a_* = 0.1, *v_a_* = 1.57 and ω_0_ = 0.2082 in (A), (C) and (D).  For *u_a_* = 0.4, *v_a_* = 1.27 and ω_0_ = 0.2042 in (A), (C) and (E). All the other parameters are the same as in Sup. File 1. |
| Fig. 4-fig. sup. 2 | In (B)-(G), *v_p_* = 0.16 for *t* < *t_g_* and *v_p_* = 3 for *t* ≥ *t_g_*. (B), (C) and (E) *t_g_* = 270 min. (D) *t_g_* = 180 min. (F) *t_g_* = 360 min. (G) *t_g_* = 450 min. All the other parameters are the same as in Sup. File 1. |
| Fig. 4-fig. sup. 3 | Parameter values are same as in Fig. 4E, F. |
| Fig. 4-fig. sup. 4 | (C) *s_r_* = 0.0064 and (D) *s_r_* = 0.0192. In both (C) and (D), *r*_0_ = 30 μm. All the other parameters are the same as in Sup. File 1. |
| Fig. 4-fig. sup. 5 | *κ_s_* = 0.06/780, and *κ*_0_ = 0.035. All the other parameters are the same as in Sup. File 1. |
| Fig. 4-fig. sup. 6 | Parameter values are the same as in Fig. 4 E, F. |
| Fig. 4-fig. sup. 8 | (B) and (D) Parameter values are the same as in Fig. 4 E, F. |
